# Spatiotemporal characteristics and influencing factors of grain yield at the county level in Shandong Province, China

**DOI:** 10.1038/s41598-022-14801-x

**Published:** 2022-07-14

**Authors:** Huanhuan He, Rijia Ding, Xinpeng Tian

**Affiliations:** 1 School of Management, China University of Mining and Technology (Beijing), Beijing, 100083 China; 2 CAS Key Laboratory of Coastal Environmental Processes and Ecological Remediation, Yantai Institute of Coastal Zone Research, Chinese Academy of Sciences, Yantai, 264003 China

**Keywords:** Environmental sciences, Environmental social sciences

## Abstract

China’s food security has always been a high priority issue on the political agenda with rapid urbanization affecting agricultural land, and it is challenged by several factors, such as human activities, social politics and policy. Shandong is an important grain-producing province and the second most populous province in China. In this paper, the spatiotemporal characteristics of grain yield and their potential influencing factors were explored at the county level in Shandong by using panel data over a 19-year period. The location Gini coefficient (L-Gini) and exploratory spatial data analysis (ESDA) were used to study the spatial agglomeration characteristics of grain yield, and spatial regression methods (SRMs) were used to analyse the influencing factors. The results indicated that grain yield increased from 38.3 million metric tons to 53.2 million metric tons in 2000–2018, with a growth rate of approximately 28.0%. The increase in grain yield in Shandong was due to the driving effect of radiation from high-yield counties to surrounding moderate-yield counties. This revealed an upward trend of spatial polarization in Shandong’s grain yield. In 2000–2018, the L-Gini and global Moran’s I increased from 0.330 to 0.479 and from 0.369 to 0.528, respectively. The number of counties in high-high (HH) and low-low (LL) agglomeration areas increased, and the spatial polarization effect was significant. SRMs analysis showed that irrigation investment and non-grain attention have significant positive and negative effects on grain production, respectively. The spatial relationship between grain yield and its influencing factors was explored to provide a reference for formulating scientific and rational agricultural policies.

## Introduction

Food security has been a significant worldwide concern in terms of human survival and development^1^. China is the world's largest agricultural producer, with a quarter of the total global grain yield. However, with a population of over 1.4 billion, it has officially been the world's largest agricultural importer since 2019^2^. Over the past decades, China has experienced rapid industrialization and urbanization, resulting in the sacrifice of many high-quality farmlands^3,4^. Land use competition between urbanization and agriculture poses a major challenge to maintaining food security, and this will continue to exist in the coming decades^5–7^.

The Chinese government and people have made marked contributions to the UN’s Millennium Development Goals (MDGs) in their efforts to reduce food insecurity^8^. On February 22, 2021, China released the No.1 Central Document, which ushered in the country's 18th consecutive year focusing on “three rural issues”, namely, agriculture, rural areas, and farmers. This document proposed that governments at all levels shoulder political responsibility for food security. Over the past 17 years, China's grain yield has maintained sustained and stable growth, and the total grain yield has remained above 650 billion kg in recent years. However, food security is still a great challenge due to a shrinking rural labor force, the reduction of available farmland due to urban development, climate change and water shortage^9–12^. In addition, COVID-19 had certain impacts on agricultural production and food supply chains, which pose a threat to food security^13,14^.

The problems of land use change and farmland management systems are particularly prominent in Shandong. However, Shandong is one of the 13 grain-producing provinces and the largest grain-export province in China. Therefore, what happens to Shandong’s agricultural production affects the national food security and social stability, especially with regards to corn and wheat. The grain status of Shandong proves that it is reasonable to choose this area as the research area.

Fluctuations in grain yield are related to various potential drivers, such as technological advances^15^, nitrogen fertilization^16^, irrigation^17^, the agricultural labor force^18^, agricultural mechanization^19^ and urbanization development^20^. Pan et al. analyzed the spatial pattern changes in China's grain output and investigated the potential driving factors of grain yield using SRMs, and the results showed that implementing large-scale mechanization and improving planting technology and mechanization level are effective ways to ensure grain yield increases^16^. Shi et al. studied food security on the Loess Plateau of China, and the results showed that the improvement of agricultural conditions, such as irrigation and fertilization, will increase the unit yield of crops by 20%^21^. Zhang et al. used the C-D production function model to measure the impact of various driving factors on grain yield and found that planting area, pesticide application, effective precipitation and chemical fertilizer have a positive impact on corn yield^22^.

Previous studies on the relationship between grain yield and influencing factors mainly focused on the national and provincial levels, and few took the county level as the research scale. Shandong is a province with unbalanced development. There are obvious differences in natural and economic conditions among counties, which may lead to differences in grain yield in different areas. Therefore, the study of grain production change at the county level in Shandong Province can evaluate its temporal and spatial characteristics more accurately, which is helpful to improve the accuracy of the results. In this context, this study aims to: (1) study the spatiotemporal dynamics of grain yield at the county level in Shandong Province, (2) analyse the spatial correlation and heterogeneity of grain yield between counties using the locational Gini coefficient and global and local Moran’s I, (3) explore the spatial dependence of grain yield and its potential influencing factors using spatial econometric models, and (4) propose reasonable suggestions according to the spatial correlation and influencing factors of grain yield.

## Materials and methodology

### Study area

**Figure 1:**
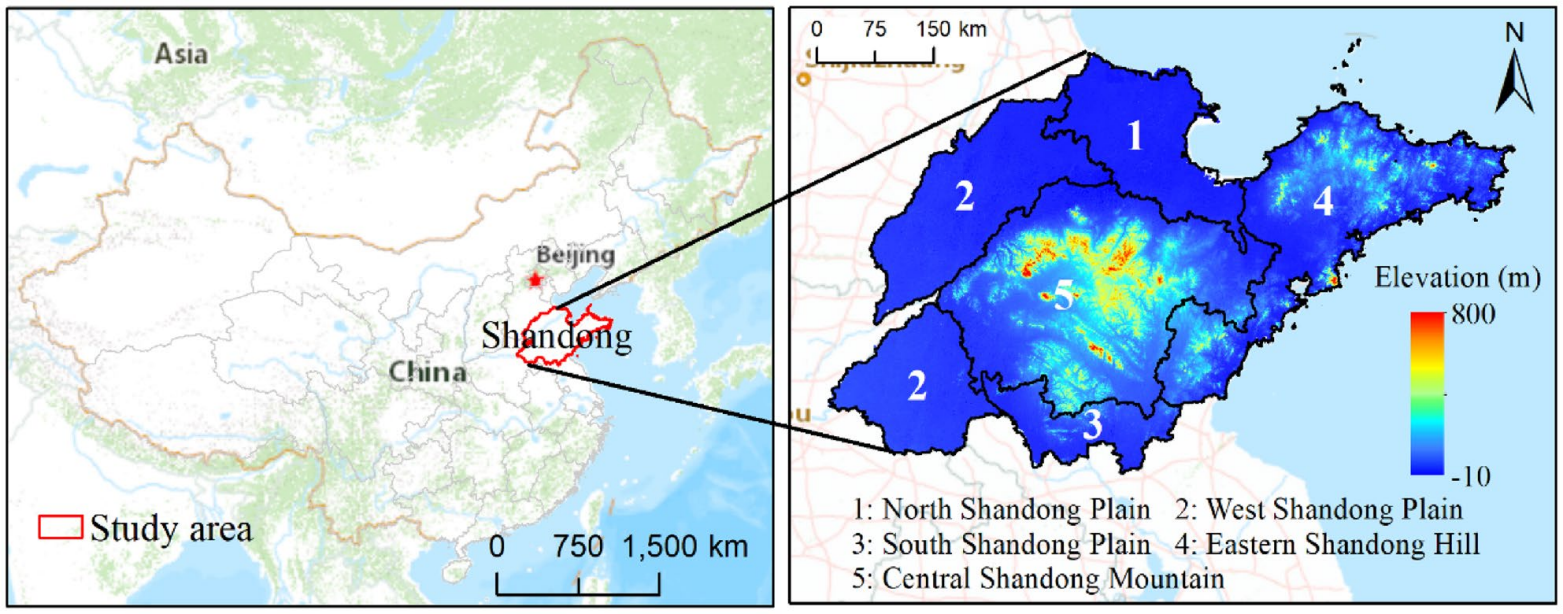
Geographical location and elevation (30-m resolution) of the study area. Basemap used with permission from ESRI China Online Community (https://map.geoq.cn/ArcGIS/rest/services/ChinaOnlineCommunity/MapServer). The elevation data were obtained from USGS Earth Explorer (https://www.earthexplorer.usgs.gov)^23^. Maps generated in ArcMap 10.2 (https://support.esri.com/zh-cn/products/desktop/arcgis-desktop/arcmap/10-2-2).

The research area for this study is Shandong Province, located in the northernmost region of the lower Yellow River in eastern China, and it borders the Yellow Sea and the Bohai Sea (114° 19′–122° 43′ E, 34° 22′–38° 15′ N), as shown in Fig. 1. The area includes 16 cities and 137 counties. The whole province covers an area of over 155,800 km^2^, with plains, hills and mountains areas accounting for 65.6%, 15.4% and 14.6%, respectively. The northern, western, and southern parts of the province are part of the vast North China Plain. The east is the hilly Shandong Peninsula and the centre of the province is more mountainous. Shandong has a warm temperate monsoon climate with four distinct seasons and a mild climate. The average annual temperature ranges from 11 to 14 °C for the entire province, and the average annual precipitation ranges from 550 to 950 mm. As 60% of the precipitation occurs in summer, the province is prone to waterlogging in summer and drought in winter and spring, which has a significant impact on agriculture. Shandong is an important agricultural area in China. It possesses 6% of China's arable land and produces 8% of China's grain. The grain-producing areas are mainly distributed in the Southwest Plain, the Northwest Plain and the Jiaolai Plain.

### Data sources and processing

In this study, the multisource statistical data of 137 counties in Shandong from 2000 to 2018 were obtained from the provincial and municipal statistical yearbooks. These datasets were derived from the following sources: (1) The county-level grain yield, grain sown area, cultivated area, total agricultural machinery power, effective irrigation area, agricultural labor force and consumption of chemical fertilizer precipitation datasets were collected from the Statistical Yearbook of Shandong Province (http://tjj.shandong.gov.cn). For years without data, data were obtained from municipal statistical yearbooks. (2) The urbanization rate, the proportion of primary industry in GDP and the proportion of non-grain attention were calculated according to the data in the statistical yearbook of each city (2001–2019) (http://qdtj.qingdao.gov.cn et al.). (3) The county-level administrative division data were obtained from the Database of Global Administrative Areas (GADM) version 4.0.4 database (https://www.gadm.org). These data were modified according to the adjustment of administrative divisions. The descriptive statistics of these variables are presented in Table 1.

**Table 1:** Descriptive statistics of all variables, 2000–2018.

Variables	Indicators	Mean	Standard deviation	Minimum	Maximum	Index description	Direction
Grain yield, GY	Total grain yield (10^4^ t)	276.1	171.0	21.3	764.3	The grain yield is related to food security, and each potential driving factor is related to the total grain yield	/
Grain-sown area, GSA	Total grain crops area (10^3^ hm^2^)	26.4	132.9	0.1	1,191.0	Grain-sown area is the basic condition to ensure the scale of grain yield^24^	Positive
Irrigation investment, II	Effective irrigation area (10^3^ hm^2^)	290.9	155.0	36.7	646.5	Irrigation plays an important role in agricultural production^25^, as Shandong is experiencing water scarcity	Positive
Mechanization investment, MI	Total agricultural machinery power (10^4^ kW)	606.7	355.7	68.1	1,523.0	The popularization of agricultural mechanization can enable the engagement in large-scale GP with less labor^26^ and promote the scale of GP	Positive
Fertilizer investment, FI	Consumption of chemical fertilizers (10^3^ t)	833.8	426.7	119.0	1,675.0	Reasonable fertilizer is necessary to keep grain yield stable and increasing^27^. It plays a major role in promoting crop growth and increasing grain yield^28^	Positive
Agricultural labor force, ALF	Number of people in the agricultural labor force (10^4^ people)	232.6	123.1	44.6	575.5	Agricultural labor force is the basic factor of agricultural production^29^, especially in Shandong, where the level of agricultural mechanization is low	Positive
Non-grain attention, NGA	Proportion of non-grain crop area to the total cultivated area (%)	33.7	9.3	10.0	64.0	To increase income, farmers often increase the planting proportion of cash crops, such as cotton and oil. The adjustment of planting structure can affect the grain sown area and grain yield	Negative
Urbanization rate, UR	Proportion of urban population to total population (%)	42.9	15.9	5.0	81.0	The migration of the rural population to cities has reduced the agricultural labor force but also improved the level of agricultural mechanization^30^	Unknown
GDP	Gross domestic product (10^4^ yuan)	2,249.0	1,944.0	110.4	12,002.0	The change of GDP may be the result of planting structure adjustment, and its increase can in turn provide economic security for agricultural production	Unknown
GPC	GDP per capita (yuan/person)	4.4	3.4	0.2	19.2	Same as GDP	Unknown
Agricultural economy, AE	Proportion of the primary industry in GDP (%)	10.9	6.7	2.9	50.2	The increased GDP of the primary industry can be used as an indicator of agricultural production	Unknown
Precipitation, PR	Average annual precipitation (mm)	686.7	199.8	275.9	1,353.0	Agricultural success depends on the efficient use of precipitation, especially in non-irrigated cropping systems^31^	Positive

### Methods

#### Spatial difference analysis

At present, the most common measurement method for equilibrium analysis in the study of spatial differences is the locational Gini coefficient (L-Gini), which was proposed by Krugman^32^. The L-Gini is a variant of the Gini coefficient. Initially, it was used as a measure indicator to compare the concentration of the geographical distribution of an industry. The spatial distribution of grain yield shows diversity in China^16^. Therefore, in this study, the L-Gini represents the degree of agglomeration of grain yield on a county scale. The specific calculation process is shown as follows:

1 $$L-Gini= \frac{1}{2(n-1)}{\sum }_{i=1}^{n}{\sum }_{j=1}^{n}\left|{x}_{i}-{x}_{j}\right|$$

where *n* is the number of counties in Shandong, and *x*_*i*_ and *x*_*j*_ represent the proportion of the grain yield of counties *i* and *j* in the total grain yield of the whole province, respectively. The L-Gini value ranges from 0 to 1; the closer the coefficient is to 1, the higher the degree of spatial geographical agglomeration of industries**,** and the closer the coefficient is to 0, the more uniform the spatial distribution of the industry^33,34^.

#### Exploratory spatial data analysis (ESDA)

Exploratory spatial data analysis (ESDA) focuses on identifying the spatial dependence and heterogeneity of georeferenced data, which was developed as an extension of exploratory data analysis (EDA)^35,36^. The various techniques of ESDA have been developed, and ESDA tools have become much more available with efforts integrating geographic information science (GIS) and spatial data analysis^37,38^. Over the last ten years, specialized stand-alone ESDA software packages have been developed, such as GeoVISTA^39^ and GeoDa^40^.

In this study, some of the statistical data used were from geography, and the spatial correlation should not be ignored. ESDA includes the global and local spatial autocorrelation coefficients, namely, global Moran's I and local Moran’s I respectively. Global Moran’s I is a global statistic that provides overall characteristics about the spatial distribution of variables^41^. It is calculated as:

2 $$I= \frac{n{\sum }_{i=1}^{n}{\sum }_{j=1}^{n}{w}_{i,j}\left({x}_{i}-\overline{x }\right)\left({x}_{j}-\overline{x }\right)}{{\sum }_{i=1}^{n}{\sum }_{j=1}^{n}{w}_{i,j}{\sum }_{i=1}^{n}{\left({x}_{i}-\overline{x }\right)}^{2}}, \quad j\ne i$$

where $\overline{x }$ is the mean of the grain yield and ${w}_{i,j}$ is the spatial weight matrix used to measure the spatial connectivity between counties *i* and *j*. The value of global Moran’s I ranges from -1 to 1. If *I* > 0, there is a positive spatial correlation; conversely, if *I* < 0, it indicates a negative spatial correlation. If the absolute value is close to 0, the spatial distribution is random.

Local Moran's I is relatively similar to global Moran's I, as shown below, it is a measure of the difference between the observed value *i* and the average multiplied by the sum of the differences between its adjacent values and the average^42^. The local Moran’s I of county *i* (${I}_{i}$) is calculated as follows:

3 $${I}_{i}= \frac{n\left({x}_{i}-\overline{x }\right)}{{\sum }_{j=1}^{n}{\left({x}_{j}-\overline{x }\right)}^{2}}{\sum }_{j=1}^{n}{w}_{i,j}\left({x}_{j}-\overline{x }\right)$$

#### Spatial regression methods

The spatial regression method (SRM) can consider the correlation between observations, which is usually generated when collecting observations from points or regions in space^43^. The basic models of SRMs include the spatial lag model (SLM) and spatial error model (SEM), which introduce a spatial weight matrix on the basis of the ordinary least squares (OLS) model, describing the structure of the dependence between the units in the sample^44^. Therefore, SRMs are able to reflect the regional correlations and differences in the panel data. In this study, SRMs were used to explore the spatial dependence between grain yield and each potential driving factor, that is, to quantitatively evaluate the impact of various factors on grain yield change. The SLM and SEM are expressed by Eqs. (4) and (5).

4 $$Y= \rho WY+X\beta +\varepsilon$$

5 $$Y= X\beta +\lambda W\mu +\varepsilon =X\beta +{\left(1-\lambda W\right)}^{-1}\varepsilon$$

where *Y* is an *N* × 1 dependent variable vector (i.e., standardized grain yield value), *X* represents the *N* × *K* matrix of the *K* explanatory variables (i.e., potential driving factors), $\rho$ represents the coefficient of the spatially lagged dependent variable, *W* denotes a spatial weights matrix (*N* × *N*) based on the spatial adjacency relation of the research unit, $\beta$ is the spatial regression coefficient, $\lambda$ is a coefficient on the spatially correlated errors, $\mu$ is the disturbance term, which has its own spatial autoregressive structure, and $\varepsilon$ is the error term which is normally distributed with an error variance matrix.

## Results and analysis

### Spatiotemporal patterns of grain yield

**Figure 2:**
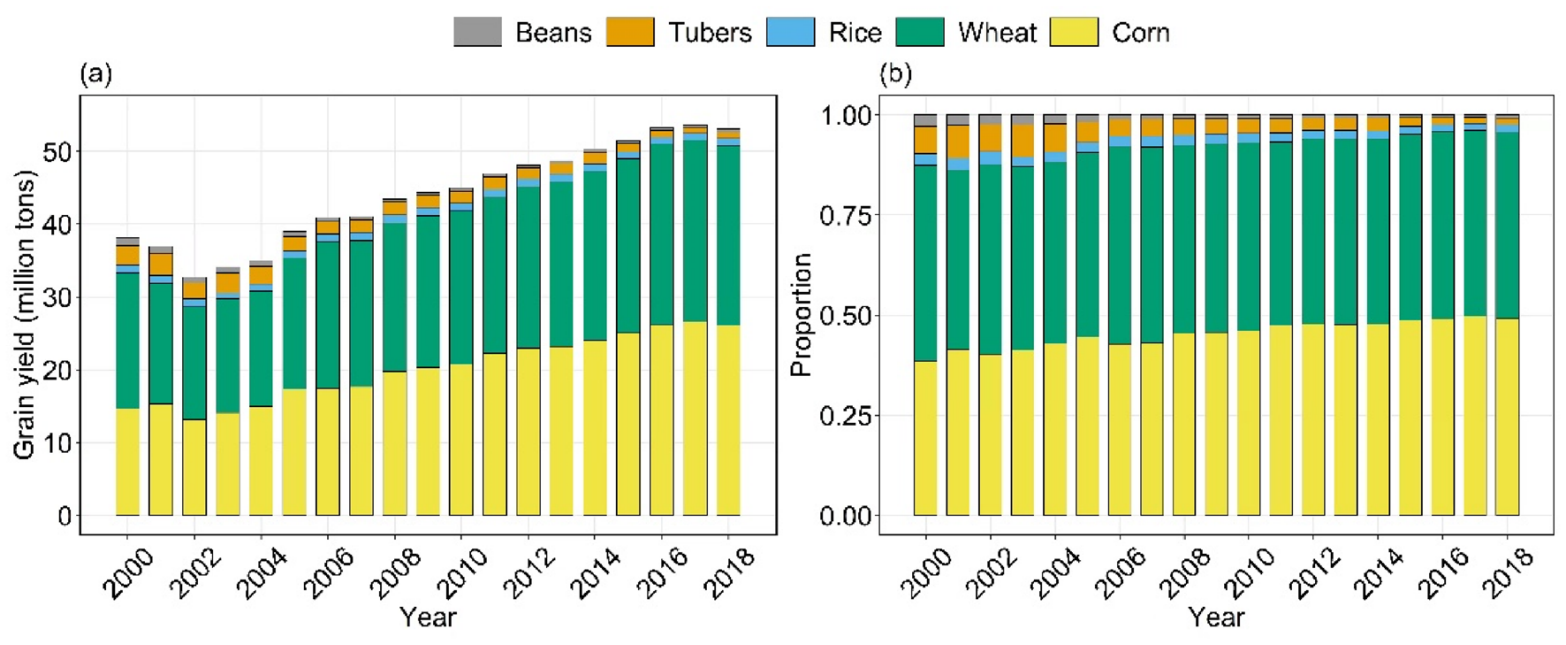
The temporal evolution of the (**a**) yield and (**b**) proportion of corn, wheat, rice, tubers and beans from each year during 2000 and 2018. The data were collected from the statistical yearbook of Shandong Province published by the National Bureau of Statistics of China (https://data.stats.gov.cn/).

In this study, the grain crops include cereal (corn, wheat and rice), beans and tubers according to Chinese statistical data. This is different from FAO (Food and Agriculture Organization) statistics and the World Bank, which refer to cereal grains (such as corn, wheat, rice, barley, millet and sorghum) only. Figure 2 depicts the temporal evolution of the yield and proportion of grains in Shandong from 2000 to 2018. In the first stage (2000–2002), the total grain output showed a downward trend from 38.4 to 32.9 million tons, where the lowest value appeared in 2002. In the second stage (2003–2018), the total grain output still showed a rapid growth trend and increased steadily for 15 consecutive years (2003–2017), and the average annual growth rate of grain yield was approximately 3.4%. It is worth noting that grain yield tended to be stable (at the level of 53 million tons) from 2016 to 2018, with a small decline in 2018. Corn and wheat accounted for more than 85.7% all year and nearly 96.0% of the total grain yield in recent years. The proportion of other crops decreased to varying degrees, especially tubers, from 8.1% in 2001 to 1.5% in 2017.

**Figure 3:**
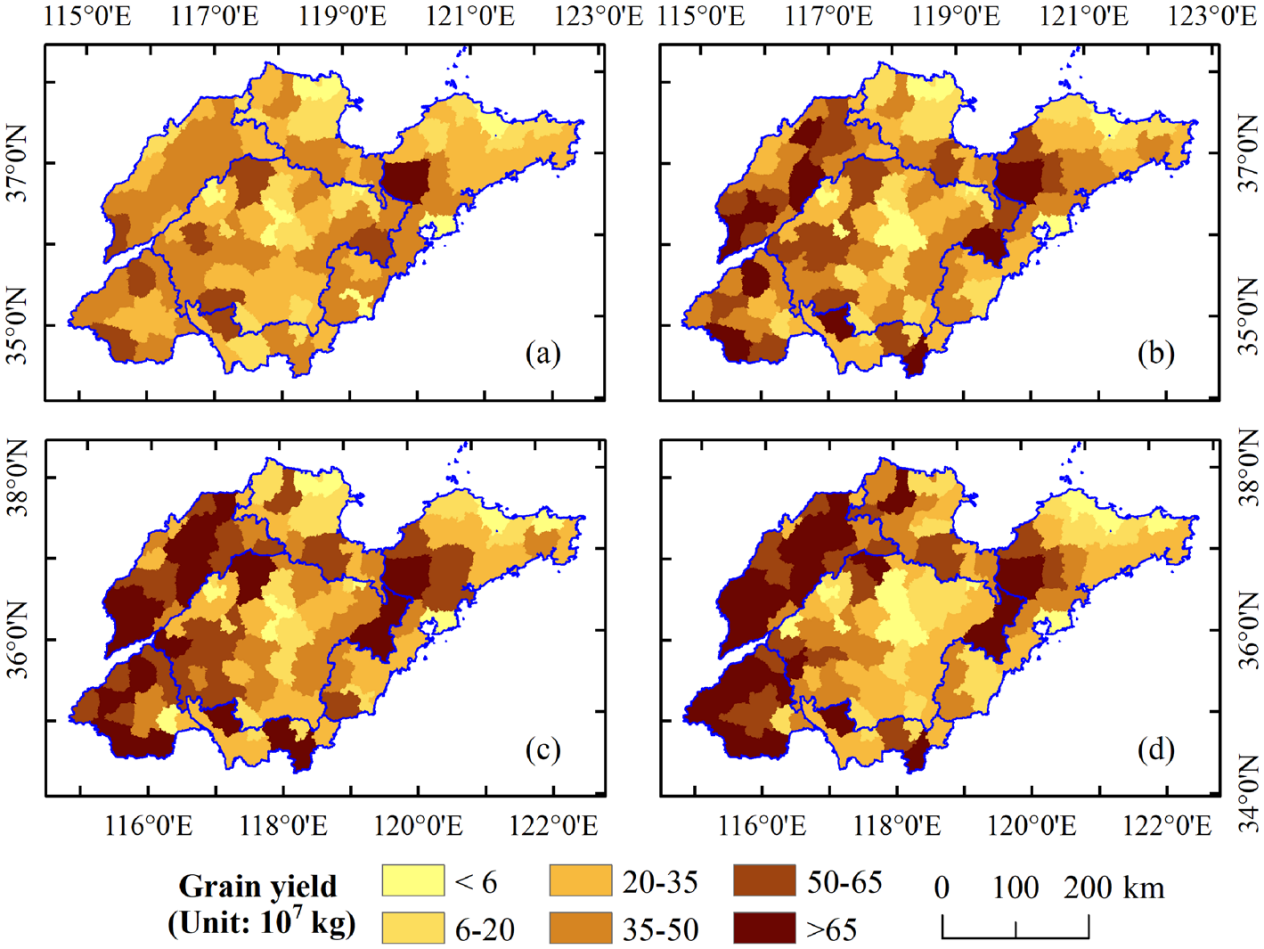
Spatial patterns of the county-level grain yield of Shandong in (**a**) 2000, (**b**) 2006, (**c**) 2012, and (**d**) 2018. The data were extracted from the Shandong Statistical Yearbook (2001–2019). The polygon shapefile of the study region was obtained from GDAM Version 4.0.4 (https://www.gadm.org/). Maps generated in ArcMap 10.2 (https://support.esri.com/zh-cn/products/desktop/arcgis-desktop/arcmap/10-2-2).

**Figure 4:**
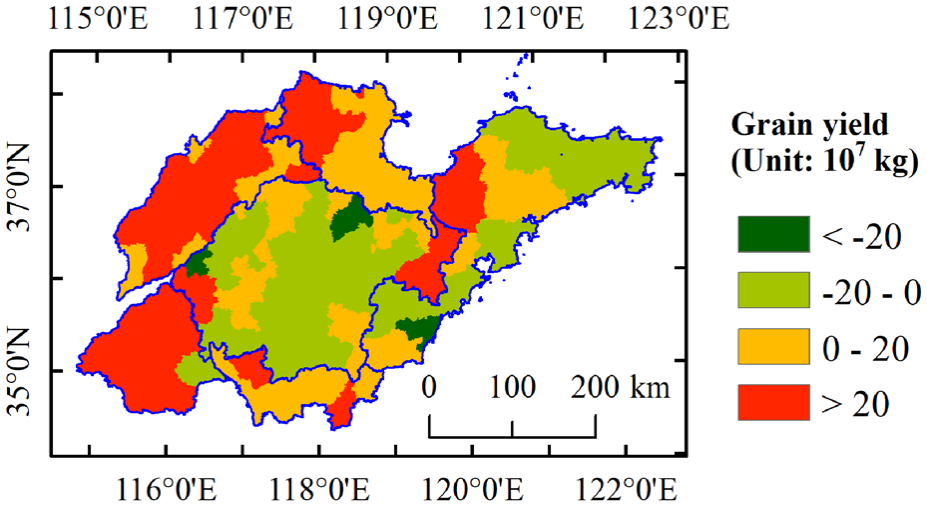
The difference in grain yield between 2018 and 2000. The polygon shapefile of the study region was obtained from GDAM Version 4.0.4 (https://www.gadm.org/). Maps generated in ArcMap 10.2 (https://support.esri.com/zh-cn/products/desktop/arcgis-desktop/arcmap/10-2-2).

The spatial patterns of Shandong’s grain yield at the county level in 2000, 2006, 2012 and 2018 were shown in Fig. 3a–d, respectively. The Geo-visualization shows that the total grain output shows an upward trend, with a large difference in the spatial distribution of increasing areas. The grain yield was divided into six categories and five levels: very low (< 6), low (6–20), moderate (20–35 and 35–50), high (50–65) and very high (> 65) grain yield. The spatial distribution of the grain yield level is continuous. High-yield counties were mainly located in the plains of western Shandong. Furthermore, the average annual growth rate of grain yield in the Shandong west plains region is 5.6%, which is higher than those of the other plain regions, such as the Shandong north plain and Shandong south plain, which are 2.3% and 2.1%, respectively. Figure 4 shows the difference in grain yield between 2018 and 2000. The counties where grain yield increased by more than 20 × 10^7^ kg were mainly distributed in the Shandong west plain region. The grain yield in the Shandong north plain and Shandong south plain also increased, mostly in the range of 0–20 × 10^7^ kg. However, in the eastern Shandong hills and central Shandong mountainous regions, the grain yield showed a downward trend, especially in the latter region, and the yield reduction range of most counties was between -20 and 0 × 10^7^ kg. From the perspective of changes in the spatial distribution of high-yield counties, the counties adjacent to high-yield areas are more likely to be transformed into high-yield counties, especially in traditional agricultural areas in inland Shandong Province.

**Figure 5:**
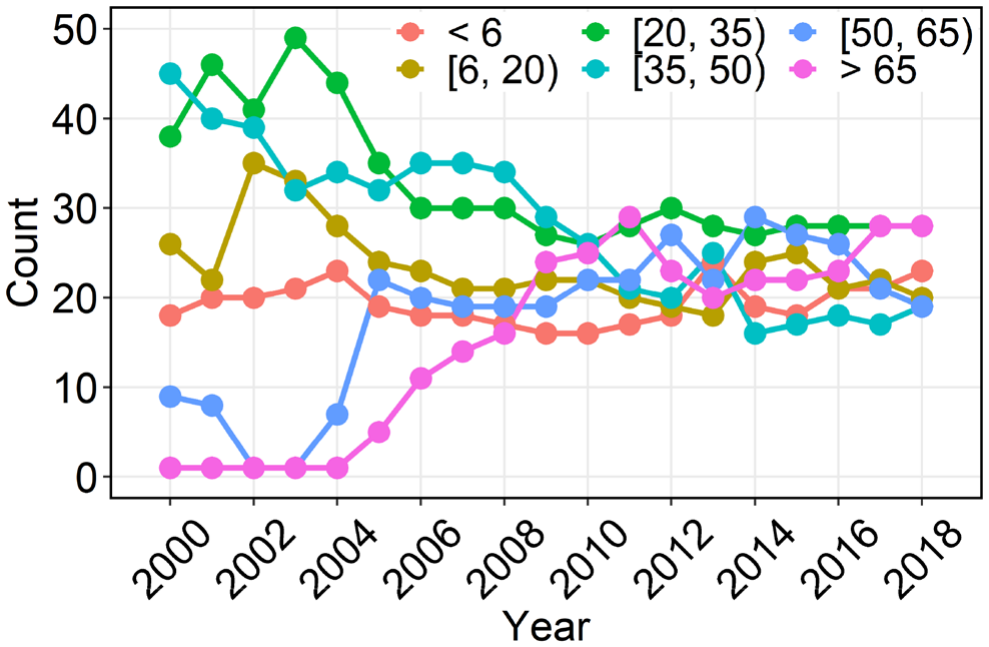
Time series of the number of county-level grain yields in six categories from 2000 to 2018.

Before 2006, Shandong was dominated by moderate-yield counties, and the number of very high- and high-yield counties was very small (Fig. 5). In approximately the last ten years, with the development of agricultural technology and the emphasis on agricultural production, the number of very high-yield and high-yield counties has increased, from 1 and 9 in 2000 to 28 and 19 in 2018, respectively. In contrast, the number of moderate-yield counties showed a downward trend during the study period. The number of very low-yield and low-yield counties did not change significantly, with approximately 20. These results indicate that the increase in grain yield in Shandong was mainly due to the driving effect of the radiation from high-yield counties to the surrounding moderate-yield counties.

### Evolution of agglomeration and autocorrelation degree of grain yield

**Figure 6:**
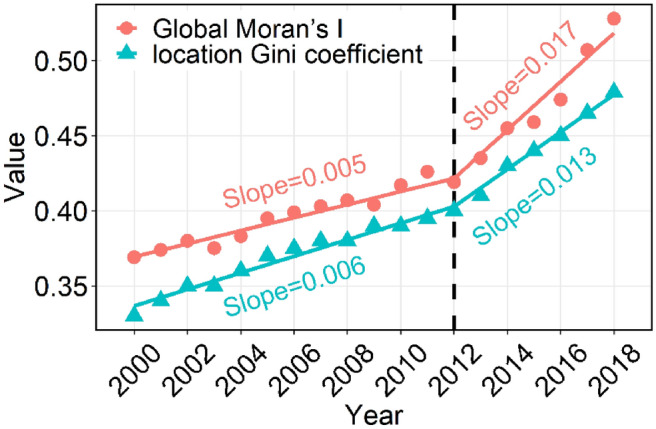
Trend of the location Gini coefficient and global Moran’s I of grain yield from 2000 to 2018.

In this study, the L-Gini coefficient and global Moran’s I were calculated for grain yield in all years. These two indicators can identify whether there is a spatial agglomeration in Shandong’s grain yield at every stage and quantitatively express the intensity of the spatial agglomeration. As shown in Fig. 6, the change trends of the L-Gini and global Moran’s I of grain yield showed a similar tendency. Before 2012, the L-Gini and global Moran’s I showed an upward trend of slow fluctuation. In this stage, the L-Gini $\in$ [0.3, 0.4) indicates that the agglomeration degree of grain yield is low. From 2012 to 2018, the L-Gini and global Moran’s I presented a rapid upward trend with slopes of 0.013 and 0.017, respectively. Moreover, the L-Gini was > 0.4, revealing that the cluster level of Shandong’s grain yield has been increasing in recent years.

**Figure 7:**
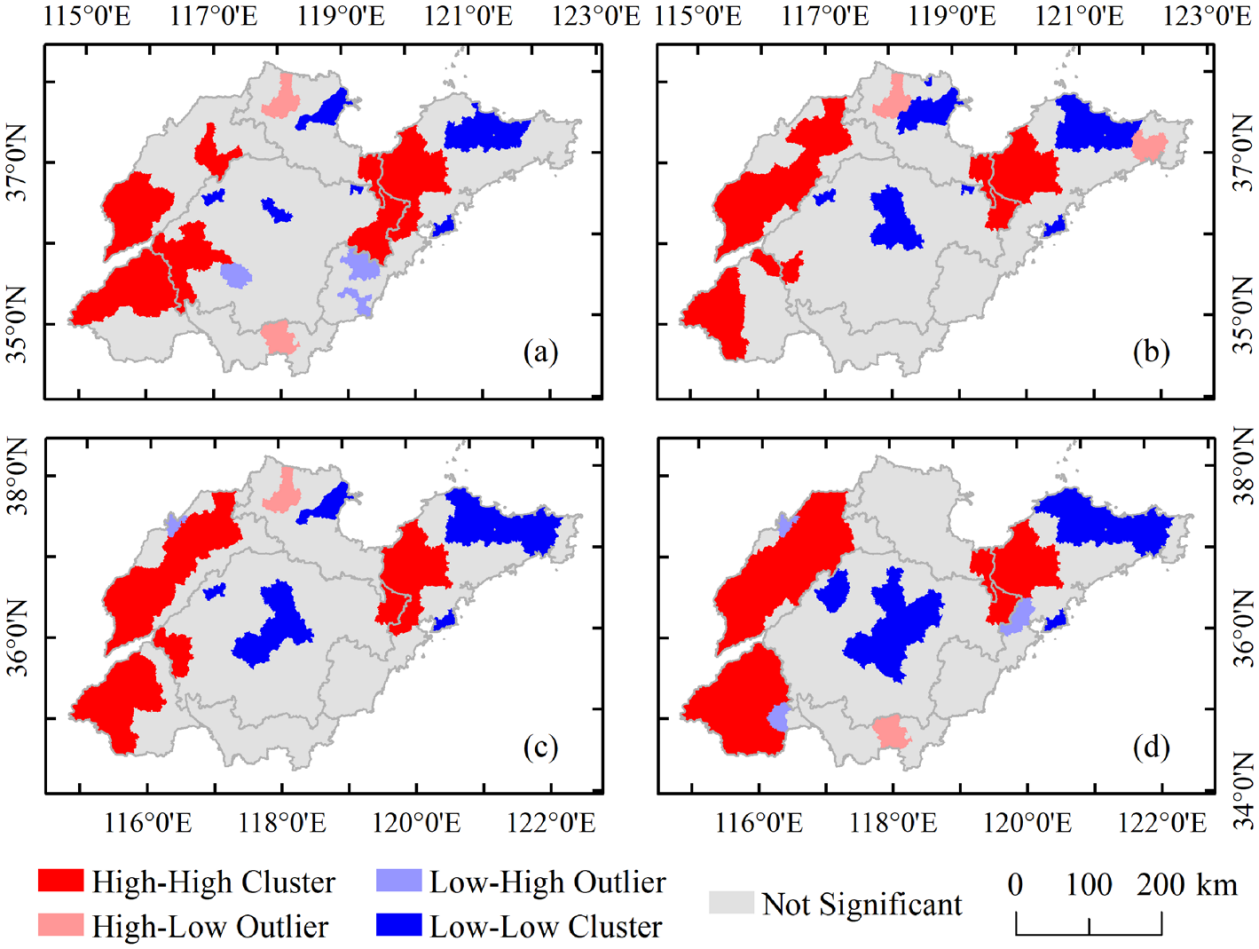
The LISA cluster map of grain yield in (**a**) 2000, (**b**) 2006, (**c**) 2012, and (**d**) 2018. The polygon shapefile of the study region was obtained from GDAM Version 4.0.4 (https://www.gadm.org/). Maps generated in ArcMap 10.2 (https://support.esri.com/zh-cn/products/desktop/arcgis-desktop/arcmap/10-2-2).

The univariate LISA cluster map shows local spatial correlation and illustrates spatial clustering and outliers. The univariate LISA results of grain yield at a certain significance (p < 0.05) level across 137 counties of Shandong from 2000 to 2018 are presented in Fig. 7. The LISA clustering map is divided into four types of geographical agglomeration types, including high-high (HH), high-low (HL), low–high (LH) and low-low (LL) types. For example, the "HH" type refers to areas with a higher-than-average yield, and their adjacent areas are also higher than average. These areas are called "hot spots"^45^. On the other hand, "HL" means that areas with grain production above the average are surrounded by areas with grain production below the average. There are obvious HH agglomeration characteristics in grain yields in the Shandong west plain and Jiaolai Plain regions, both of which are traditional agricultural areas with high levels of agricultural management. The LL clustering regions were concentrated mainly in the central mountain and north of the Shandong Peninsula regions, where there is a high urbanization level and population density. Comparing Fig. 7 with Fig. 3, it is clear that high-yield counties are also HH aggregation areas, and low-yield counties are LL aggregation areas, and grain yield and spatial autocorrelation have similar spatial evolution patterns.

### Spatial dependence of grain yield on potential driving factors

To understand the correlation between variables and avoid the problem of collinearity, the Pearson correlation analysis and the variance inflation factor (VIF) test were performed on the variables involved in SRMs. The analysis results are shown in Tables 2 and 3, respectively. From Table 2, the dependent variable has a high correlation with each independent variable, while the correlation between independent variables is low. Then, the VIF test was performed on the variables. The results are shown in Table 3. The VIF of each independent variable is less than 10, and the average VIF is 4.32, indicating that there is no serious multicollinearity between the independent variables. The next step of the regression analysis can be performed.

**Table 2:** Correlation between grain yield and potential driving factors.

Variables	GY	GSA	II	MI	FI	ALF	NGA	UR	GDP	GPC	AE	PR
GY	1.000											
GSA	0.201***	1.000										
II	0.907***	0.159***	1.000									
MI	0.888***	0.103*	0.178***	1.000								
FI	0.838***	0.065	0.112***	0.244**	1.000							
ALF	0.772***	0.107*	0.276**	0.425	0.279	1.000						
NGA	− 0.432***	− 0.217*	− 0.219**	− 0.315**	− 0.160**	− 0.190	1.000					
UR	− 0.181**	0.199**	− 0.071***	− 0.115**	− 0.319*	− 0.224	− 0.265**	1.000				
GDP	0.194***	0.242**	0.130**	0.282**	0.122**	0.041**	− 0.171**	0.432*	1.000			
GPC	− 0.247***	0.154***	− 0.288***	− 0.144*	− 0.370	− 0.304*	− 0.134*	0.284*	0.182**	1.000		
AE	0.273***	− 0.086	0.355*	0.291**	0.253*	0.063*	0.209	− 0.089**	− 0.080***	− 0.115***	1.000	
PR	− 0.131*	0.096*	− 0.195***	− 0.184***	− 0.085	0.029	0.097*	− 0.012	− 0.044	− 0.040	− 0.072	1.000

*σ*^2^ denotes residual variance; *, **, *** represent significance at 10%, 5% and 1%, respectively.

**Table 3:** VIF test of potential driving factors.

Variable	VIF	1/VIF
FI	8.92	0.112
II	8.80	0.114
MI	5.69	0.176
ALF	5.29	0.189
GDP	4.60	0.217
UR	4.22	0.237
GPC	3.79	0.264
AE	2.40	0.416
NGA	1.38	0.723
GSA	1.22	0.820
PR	1.19	0.839
Mean VIF	4.32	

The OLS, SLM and SEM models were used to analyze the grain yield and its potential driving factors. Table 4 shows the spatial correlation of grain yield with potential driving factors. However, Moran’s I values are high and show an upward trend from 2000 to 2018 (Fig. 6), therefore, the OLS regression may be misleading. Compared with the OLS regression, SRMs show higher R^2^ and log likelihood values, especially SLM regression. Moreover, the Lagrange multiplier (LM) test shows that LM (lag) or LM (error) is highly significant, and LM (lag) robustness is also highly significant. Therefore, the SLM model is more suitable for examining the relationship between food production and its potential driving factors.

**Table 4:** Estimation results of the OLS, SLM and SEM regressions between grain yield and each potential driving factor across periods.

Variables	OLS	SLM	SEM
GSA	0.073**	0.076**	0.109***
II	0.586***	0.498***	0.540***
MI	0.331***	0.134***	0.266***
FI	0.180***	0.352***	0.071
AIL	0.212***	0.199***	0.228***
NGA	− 0.251***	− 0.231***	− 0.238***
UR	− 0.039	− 0.015	0.051
GDP	− 0.138***	− 0.103***	− 0.054
GPC	− 0.076*	− 0.175***	− 0.208***
AE	0.261***	0.442***	0.193***
PR	0.040**	0.004	0.007
Constant	0.182		
*ρ*		0.213	
*λ*			0.371
R^2^	0.726	0.863	0.826
corr-R^2^	0.701	0.827	0.791
*σ*^2^	0.004	0.001	0.001
Log likelihood	437.070	670.657	625.163
Lagrange Multiplier (lag)		31.671***	
Robust LM-Lag		18.184***	
Lagrange Multiplier (error)			15.177***
Robust LM-Error			1.691

*σ*^2^ denotes residual variance; *, **, *** represent significance at 10%, 5% and 1%, respectively.

In the SLM regression, the spatial autocorrelation coefficient *ρ* is 0.213, indicating that there is obvious spatial agglomeration in the grain yield in Shandong Province. The spatial dependence and spatial spillover effects are significant. The geographical conditions and climate environment of the neighboring areas are similar, which is conducive to the dissemination, promotion and reference of information and technology, making the development of grain production in neighboring areas appear to be similar. The results indicate that all the independent variables pass the significance test except for urbanization rate and precipitation. Among these, irrigation investment, agricultural economy, fertilizer investment, agricultural labor force and grain sown area have a positive impact on grain yield, and the impact degree decreases in turn. The estimated coefficient of irrigation investment was the maximum at 0.498, indicating that there was a strong positive effect between the effective irrigation area and grain yield. The estimated coefficients of the agricultural economy and fertilizer investment were 0.442 and 0.351, respectively, implying that agriculture was highly dependent on the agricultural economy and chemical fertilizer, which played a key role in improving grain yield. In addition, the increase in the agricultural labor force, mechanization investment and grain sown area also increase grain yield. The estimated coefficient of non-grain attention was − 0.231, that is, the proportion of non-grain sown areas had a negative impact on grain production, which is consistent with expectations. Among the total sown area of crops, the larger the proportion of non-grain sown areas, the less arable land used for growing grain, and the lower the grain yield. The regression coefficients of GDP and GPC were − 0.103 and − 0.175, respectively, showing a negative impact on grain yield. This means that the higher the regional economic level and the higher the per capita income level are, the more important the development of secondary and tertiary industries, while ignoring agricultural industries with insufficient comparative advantages leads to a decline in grain production.

## Discussion

### Spatial effect and influencing factors of grain

The results show the spatial effect of the spatiotemporal distribution of grain yield (as shown in Figs. 3 and 7). The distribution of grain yield in counties in Shandong is uneven. On the one hand, the L-Gini of grain yield increased year by year and was greater than 0.400 after 2012, revealing that the cluster level has been continuously enhanced in recent years. The high-yield counties are mainly distributed in the plain area of western Shandong; however, in the eastern Shandong hill and central Shandong mountain regions, the grain yield showed a downward trend. This is mainly because the traditional agricultural areas give more attention to agricultural production, while the more developed coastal areas give more attention to the development of secondary and tertiary industries. On the other hand, the global Moran’s I was greater than 0.369 during the study period, indicating that there was a positive spatial correlation of grain yield, and the spatial correlation gradually increased, especially after 2012. From 2012 to 2018, the L-Gini and global Moran’s I presented a rapid upward trend with slopes of 0.013 and 0.017, respectively.

The rapid development of urbanization has led to a large number of people seeking employment in developed areas; at the same time, the continuous expansion of regional extension and the occupation of a large amount of arable land have led to a continuous threat to grain production between traditional agricultural areas and economically developed areas. Due to the radiation effect, the grain yield in economically developed areas was lower, and high-yield grain areas belong to plain areas, which facilitates large-scale management; thus, the high-yield areas are further concentrated. From the LISA clustering map, the number of counties in the HH and LL agglomeration areas is relatively large, and the spatial polarization trend of the grain yield pattern continues to intensify.

In terms of influencing factors, effective irrigation, agricultural machinery power, increase in the amount of agricultural labor and fertilizer application have a positive impact on grain yield. The effect of effective irrigation area on grain yield was significantly positive, mainly because effective irrigation promotes crop growth. The regulation of droughts and floods improves the ability of cultivated land to resist natural disasters and protects and promotes grain production. On the one hand, increasing the input of agricultural machinery can save human resources; on the other hand, it can improve labor efficiency and promote the improvement of grain production efficiency^16^. In addition, the use of agricultural machinery and the spread of advanced technology in surrounding areas lead to convergence between regions, which has significant spatial spillover effects and promotes the development of regional food production. The increase in agricultural labor input makes the agricultural management mode change from extensive to intensive, thus improving the grain production capacity. Scientific and reasonable fertilization can also improve grain yield^29^.

GDP and GPC have a negative impact on grain yield; that is, the higher the regional economic level and the higher the per capita income level are, the more important the development of secondary and tertiary industries, while ignoring the agricultural industries with insufficient comparative advantages (such as Jinan and Qingdao). Due to the radiation effect of the economy, the development of the regional economy also exhibits spatial agglomeration. The spatial agglomeration effect of the economy also affects the spatial agglomeration effect of grain yield. For example, the level of economic development in the Jiaodong Peninsula area increased rapidly, while the level of grain yield relatively decreased, which has a certain impact on the grain yield in the surrounding areas. The proportion of non-grain sown areas has a negative impact on grain yield, which is consistent with expectations. Among the total sown area of crops, the larger the proportion of non-grain sown area, the less arable land is used for growing grain, and the lower the grain yield. Scientific and reasonable inputs, such as reasonable irrigation areas, the application of chemical fertilizer and the input of the agricultural labor force, can improve grain output. With the development of large-scale agricultural operations and the use of agricultural machinery, grain planting has become more scientific and efficient.

### Enlightenment of research on agricultural policy-making

The expansion of cities poses a threat to food production. Ensuring food security and sustainable agricultural production has become an important and urgent problem for agricultural decision-makers. Shandong is the main grain-producing area in China. This study considers the temporal and spatial dynamics and influencing factors of grain output in Shandong from 2000 to 2018. Based on the results, relevant suggestions are proposed to improve grain yield and ensure food security. Specific recommendations are as follows:

1. Due to the spatial autocorrelation of grain yields in various regions of Shandong Province, grain yields are affected not only by relevant factors in this region but also by neighboring regions. Therefore, full consideration should be given to the interaction and influence of interregional grain production, and emphasis should be placed on radiation-driven development. Full play should be given to the radiation and leading role of high-yield areas, and information sharing and improvements in grain production capacity should be promoted.
2. The quantity and quality of cultivated land should be protected. From the results above, the sown area of grain is positively correlated with grain yield, so it is necessary to protect the quantity and quality of cultivated land. The government should strictly abide by boundaries of cultivated land and strengthen land approval and supervision.
3. The input and use of agricultural machinery should be strengthened. Effective mechanical irrigation and the use of agricultural technology can increase grain production. Therefore, the construction of farmland water conservancy facilities should be further strengthened to accompany grain production. At the same time, it is necessary to improve the level of agricultural science and technology, strengthen the transformation of scientific and technological innovation achievements, increase the investment penetration rate of agricultural machinery, and promote the improvement of grain productivity.
4. The regional economic level has a negative impact on grain yield. Therefore, as the economy develops, it is necessary to reduce the impact on agriculture and give attention to its protection.

### Strengths and limitations

Due to the vast territory of China, there are significant differences in natural and economic conditions among the provinces, resulting in differences in grain output among them. If research is conducted on a large scale, it will cover up the internal differences between provinces, and it will be of little significance for the guidance of grain yield in Shandong Province. Analysing the change in grain production in Shandong at the county level can enable a more accurate understanding of its temporal and spatial variation characteristics and provide more targeted guidance for grain production in Shandong Province.

In addition, this study combines the knowledge of agriculture, economics, geography and other related disciplines, and the spatial interaction effect was incorporated into the econometric model to analyse the potential factors of grain yield spatial change, which improves the interpretation ability of the model. The results are more targeted for the guidance of grain yield in Shandong.

Because the data sources are mainly from statistical yearbooks, the selected influencing factor index variables are limited, and deeper influencing factors such as resource endowment and farmers' behavior at the micro level may be important influencing factors. Therefore, these factors need to be further considered and discussed in the future.

## Conclusions

This study provides valuable insights into the spatiotemporal dynamics of grain yield in Shandong. By considering spatial correlation, the interaction between grain yield and its influencing factors was explored, and the following conclusions were drawn after relevant empirical analysis.

During the study period, the total grain output showed an obvious upward trend, with marked spatial agglomeration characteristics and spatial correlation. The L-Gini and global Moran's I showed an upward trend, and the high-yield counties were mainly located in the western Shandong plain, but the grain yield in the eastern and central areas of Shandong declined significantly. The number of "HH" and "LL" agglomeration areas was relatively large and gradually increased, indicating an obvious spatial polarization effect.

This study also identified spatial effects between grain yield and potential influencing factors. Among them, effective irrigation investment, agricultural economy, fertilizer investment, agricultural labor force and grain sown area had a positive impact on grain output, and the degree of influence decreases in turn. Non-grain attention, GPC and GDP showed negative effects on grain yields. Studying the dynamic changes in the spatial and temporal distribution of grain yield and its influencing factors provides a practical reference for formulating reasonable agricultural policies.

## Data Availability

Correspondence and requests for materials should be addressed to H H–H.
